# LncRNAs: key players and novel insights into diabetes mellitus

**DOI:** 10.18632/oncotarget.19921

**Published:** 2017-08-04

**Authors:** Xiaoyun He, Chunlin Ou, Yanhua Xiao, Qing Han, Hao Li, Suxian Zhou

**Affiliations:** ^1^ Department of Endocrinology, Affiliated Hospital of Guilin Medical University, Guilin 541001, China; ^2^ Department of Endocrinology, Xiangya Hospital, Central South University, Changsha 410008, China; ^3^ Cancer Research Institute, Central South University, Changsha 410078, China; ^4^ Department of Neurology, Affiliated Hospital of Guilin Medical University, Guilin 541001, China

**Keywords:** LncRNAs, diabetes mellitus, pancreatic β cells, insulin resistance, epigenetic regulation

## Abstract

Long non-coding RNAs (LncRNAs) are a class of endogenous RNA molecules, which have a transcribing length of over 200 nt, lack a complete functional open reading frame (ORF), and rarely encode a functional short peptide. Recent studies have revealed that disruption of LncRNAs levels correlates with several human diseases, including diabetes mellitus (DM), a complex multifactorial metabolic disorder affecting more than 400 million people worldwide. LncRNAs are emerging as pivotal regulators in various biological processes, in the progression of DM and its associated complications, involving pancreatic β-cell disorder, insulin resistance, and epigenetic regulation, *etc*. Further investigation into the mechanisms of action of LncRNAs in DM will be of great value in the thorough understanding of pathogenesis. However, prior to successful application of LncRNAs, further search for molecular biomarkers and drug targets to provide a new strategy for DM prevention, early diagnosis, and therapy is warranted.

## INTRODUCTION

With the completion of the Human Genome Project (HGP) and beginning of the post-genomic era, non-coding RNA (ncRNA) had aroused great interest and attention in various areas of research. After the Nobel Prize was awarded for the discovery of small interfering RNA (siRNA) in 2006 [1], ncRNAs, including long non-coding RNAs (LncRNAs) gradually gained a dominant position in biomedical research. Thousands of LncRNAs have been discovered in the human genome over the past three years, implying that LncRNAs may play an irreplaceable role in cellular functioning. Although LncRNAs do not encode functional proteins, they are involved in many physiological processes, playing essential roles in maintaining cell proliferation and differentiation [2, 3]. Increasing evidence supports the hypothesis that LncRNAs participate in physiological and pathological processes by modulating gene expression at the epigenetic, transcriptional, and posttranscriptional levels [4, 5]. Moreover, abnormal expression of LncRNAs has been associated with a variety of human diseases, including psoriasis [6], coronary artery disease [7, 8], diabetes mellitus (DM) [9, 10], tumors [11–13], etc. However, their comprehensive bio-functions and molecular mechanisms of LncRNAs in human diseases still remain elusive. Therefore, more efforts should be made to explore the LncRNA world.

DM is a class of metabolic disorders characterized by hyperglycaemia resulting from the relatively reduced insulin secretion and occurrence of insulin resistance, which can be either due to inherited or environmental factors [14, 15]. Chronic hyperglycaemia of DM is associated with long-term damage, dysfunction and failure of multiple organ systems, especially the kidneys, eyes, blood vessels, heart, and nerves [16, 17]. DM has rapidly become a global health problem affecting more than 400 million people worldwide [18], in fact it has become the third most prevalent non-infective disease (NCD) exceeded only by cardiovascular diseases and cancer [19]. The global prevalence of DM is expected to increase from 4% in 1995 to 5.4% by the year 2025 [20], and that number of patients will reach 642 million by 2040 [21]. Currently, the countries with the largest number of diabetic patients are India, China, and United States [22]. Inspiringly, the morbidity and mortality caused by DM can be reduced by regular screening, early detection, and appropriate treatment of chronic complications [23, 24]. Therefore, there is a great urgency to prevent and treat diabetes and its associated complications. On account of the widespread use of new technologies, several LncRNAs have been proved as novel regulatory players in the molecular biology of DM, which may provide new strategies for the prevention, early diagnosis and treatment of DM. In this review, we summarize the origin and overview function of LncRNAs; highlighted the roles of LncRNAs in DM; and outlined the molecular mechanisms of LncRNAs in DM. Further, the application of LncRNAs as biomarkers in the prevention, early diagnosis, and treatment of DM has also been discussed.

### Definition and situation of LncRNAs

The Human Genome Project revealed that protein-coding genes represent less than 2% of the total genome sequence [25], the remaining greater portion of DNA sequences do not code for proteins, which are regarded as “junk DNAs” that have accumulated because of the process of evolution [26, 27]. In addition, most of the “junk DNAs” are intron DNAs in animals, and are termed as non-coding DNAs (ncDNAs) [28, 29]. Generally, based on the length of a transcript, ncRNAs are divided into two categories: small ncRNAs (fewer than 200 nucleotides) and long ncRNAs (more than 200 nucleotides) [30, 31]. With the progress of the whole-genome resequencing efforts, the latest GENCODE release (version 25) (http://www.gencodegenes.org) indicated that 75–90% of the human genome was transcribed to generate a series of LncRNAs [32, 33]. Based on previous studies, the comprehensive definition of LncRNA is as follows: LncRNAs are a subset of RNAs first found in a eukaryotic cell and have a transcribing length of 200–100000 nt; they lack a complete functional open reading frame (ORF), rarely encode a functional short peptide, and are located in either the nucleus or cytoplasm [34, 35]. LncRNAs usually function as primary transcribed or as a spliced RNA [36]. Further, most of them are similar to mRNAs with respect to structural features like poly(A) tails, 5′-caps, and promoter structure. They are transcribed by RNA polymerase II and often alternatively spliced to be finally polyadenylation [37, 38].

Interestingly, the LncRNA sequences are conserved, and they exhibit strong tissue- and cell-specific expression patterns in humans [39, 40]. LncRNAs have recently gained widespread attention over microRNAs (miRNAs) and siRNAs. With the development of RNA-seq using next-generation sequencing, a growing number of LncRNAs have been discovered and defined. The total number of functional LncRNAs thus far identified has reached 184 for human disease (http://lncrnadb.org/) [41]; nevertheless, this is only the tip of the iceberg when compared to the total number of LncRNAs predicted by bio-informatics software analysis. By elucidating the relationship between LncRNAs and disease, we can further understand the complicated and multilayer regulatory system in human, and provide novel therapeutic strategies using LncRNAs as molecular markers and potential drug targets.

### Classification and functions of LncRNAs

With advancement the sequencing technologies, especially RNA-seq technology of next-generation sequencing, an increasing number of LncRNAs have been found [42, 43]. LncRNAs have displayed characteristics that can be described as ‘three more’, namely more types, more patterns and more quantity. Although many LncRNAs have been discovered, still their origin is not completely understood. Possible origin includes gene mutations, tandem duplication events, chromatin rearrangement, retrotransposition, and insertion of a transposable element [44]. LncRNAs can be classified into five categories according to their genomic proximity with neighbouring transcripts: (1) sense strand synthesis, which are located on the sense strand of annotated transcription units; (2) antisense strand synthesis, which are located on the antisense strand of annotated transcription units; (3) intronic synthesis, which are from introns of annotated genes; (4) bidirectional synthesis, which arise from both sense and antisense directions of transcription start areas; and (5) intergenic synthesis (also known as lincRNAs), whose LncRNA transcripts are from introns of annotated genes [45, 46].

**Figure 1:**
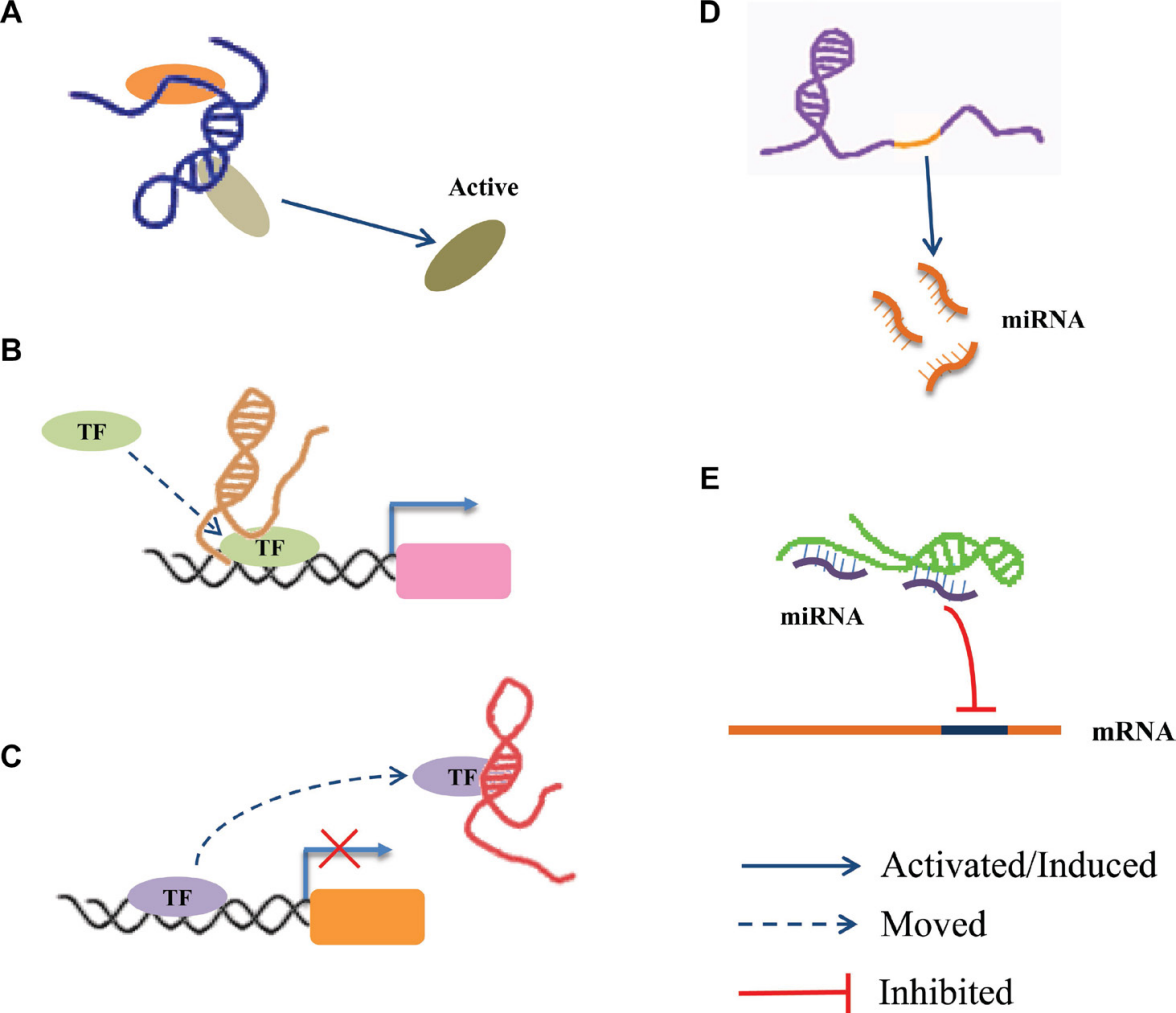
The regulatory mechanism of LncRNAs (**A**) LncRNA acts as a scaffold via recruiting and interacting with proteins and regulate the activity of proteins. (**B**) LncRNA acts as a guide to promote the gene expression via recruiting transcription factors (TF) to the region of the gene promoter. (**C**) LncRNA acts as a decoy via interacting with TF to inhibit transcriptional regulation. (**D**) LncRNA acts as host genes to promote the production of miRNA. (**E**) LncRNA serves as ceRNA to interact with miRNAs.

Accumulating evidence supports that LncRNAs are an important class of regulatory molecules in the human genome. The long nucleotide chain of LncRNAs can either form a complex spatial structure and interact with protein factors, or provide a large segment for the concurrent binding of many molecules that collectively participate in epigenetic regulation, X-chromosome silencing, genomic imprinting, nuclear and cytoplasmic trafficking, transcriptional activation and interference, mRNA splicing and degradation, *etc.* [36, 46] Although there is little knowledge about LncRNAs at present, they have been shown to have crucial roles in regulating various biological processes, such as cell proliferation, differentiation, apoptosis, senescence and death, *etc.* [47, 48]. Recently, it has been accepted that the molecular functions of LncRNAs at the epigenetic, transcriptional and post-transcriptional can be subdivided as follows (Figure 1): (1) recruiting and interacting with proteins: For example, LncRNA RMRP acts as a scaffold to interact with DEAD-box helicase 5 (DDX5) and Retinoid-related orphan receptor gamma (RORγ) to control the transcriptional program in Th17 cells [49]; (2) acting as a co-regulator or a co-repressor: For example, An antisense LncRNA was found to localize at the 5’ long-term repeats (LTR) of HIV promoter, and suppress transcription of viral genes by recruiting enhancer of zeste homolog-2 (EZH2), histone deacetylase 1 (HDAC1), and DNA-methyltransferase 3 alpha (Dnmt3a) to form a transcriptional repressor complex [50]; (3) acting as a decoy: For example, p50-associated COX-2 extragenic RNA (PACER) acts as a decoy molecule to promote the transcription of cytochrome c oxidase subunit II (COX2) in the NF-κB signalling pathway [51]; (4) acting as host genes for miRNA: For example, LncRNA H19 serves as the reservoir of miR-675-3p and miR-675-5p, which are induced during skeletal muscle differentiation [52]; (5) interacting with miRNA: For example, Long intergenic non-protein coding RNA, muscle differentiation 1 (Linc-MD1) acts as a competing endogenous lncRNA (ceRNA) to sponge miR-133 and thus regulates muscle cell differentiation in skeletal muscle [53].

### Diabetes related-LncRNA

With the rapid increase in population, aging, urbanization and the prevalence of obesity and physical inactivity, DM has become a global health problem in recent years. The development and progression of DM is a complex process that involves multiple factors and steps. Too much food and too little physical exercises are just two standard factors. The development and progression of DM is closely related to environmental factors, inherited factors, microbial infections and a decreased immune system response caused by other diseases. At present, the treatment of DM consists of five main aspects: diabetes education, self-monitoring of blood glucose, diet therapy, exercise and drug treatment. Only these five aspects of the treatment go hand in hand and can effectively control the blood glucose, thereby delaying the progress of DM. Based on pathogenesis, DM is divided into four types: type 1 diabetes mellitus (T1DM), type 2 diabetes mellitus (T2DM), gestational diabetes mellitus, and other specific types of diabetes mellitus. Amongst these, T2DM is the most common type throughout the world, exceeding 90% of all cases of diabetes [54]. T2DM is mainly influenced by lifestyle factors and genetic components [55]. The development of DM is accompanied by a series of complications, such as diabetic nephropathy (DN), diabetic retinopathy (DR), diabetic cardiomyopathy (DCM) and diabetic neuropathic pain (DNP).

LncRNAs, as multifunctional molecules, have recently gained widespread attention. Tremendous amount of data indicate that the DM susceptibility loci is associated with abnormal expression of LncRNAs [56–73], and part of these LncRNAs play a crucial role in the progression of DM and its associated complications [73–77], involving in β islet cell disorders, insulin resistance and epigenetic regulation, *etc*. However, our understanding about LncRNAs and their role in DM is still in its infancy. Through Literature search was performed in PubMed, Embase, and Web of Science with the following search terms: “Diabetes Mellitus” or “Diabetes” or “Diabetic” AND “Long Noncoding RNA” or “ LncRNA” or “ Long Non-Coding RNA” or “Long Non Protein Coding RNA” or “Long Untranslated RNA” or “Long Intergenic Non Protein Coding RNA” or “LincRNAs”. After excluding meta-analysis, letters, comments, case reports, reviews and duplicate publications, a total of 132 papers were found on the relations of LncRNAs with DM by the end of March 28, 2017. The number of relevant publications showed an explosive increase since 2012, indicating increasing focus of researchers on the relationship between DM and LncRNAs. By integrating the published literature and analysing the LncRNA Disease database (http://www.cuilab.cn/lncrnadisease) and LncRNAWiki database (http://lncrna.big.ac.cn/index.php/Main_Page) we found that till date, a total of twenty-eight LncRNAs have been associated with DM. Amongst these nineteen have been detected in human DM. An overview of the human lncRNAs that are associated with DM is shown in Table 1.

**Table 1:** LncRNAs are associated with human diabetes mellitus

LncRNAs Name	RNA Description	Aliases	Genomic Location	Diabetes Type	Dysfunction Type	Ref.
HYMAI	Hydatidiform mole associated and imprinted	NCRNA00020	6q24.2	Transient neonatal diabetes	Expression	[67]
CDKN2B-AS1	Cyclin dependent kinase inhibitor 2B (CDKN2B) antisense RNA 1	ANRIL,DKN2BAS,CDKN2B-ASC, T12,p15AS NCRNA00089PCA	9p21.3	Diabetes	Mutation	[68]
					Expression	[69]
				Type 2 diabetes	Locus/Mutation	[70]
					Mutation	[71–73]
IGF2-AS	Insulin like growth factor 2 antisense RNA	IGF2-AS1, IGF2AS, PEG8	11p15.5	Type 1 diabetes	Mutation	[74]
LINC00271	Long intergenic non-protein coding RNA 271	C6orf217, NCRNA00271	6q23.3	Type 2 diabetes	Mutation	[75]
MALAT1	Metastasis associated lung adenocarcinoma transcript 1	HCN, LINC00047, NCRNA00047NEA,T2 PRO2853	11q13.1	Diabetes	Regulation	[85]
MEG3	Maternally expressed 3	FP504, GTL2, LINC00023, NCRNA00023, PRO0518,PRO2160,	14q32.2	Type 1 diabetes	Locus	[76]
PDZRN3-AS1	PDZ domain containing ring finger 3(PDZRN3) antisense RNA 1	N/A	3p13	Type 2 diabetes	Mutation	[77]
PTV1	Plasmacytoma variant translocation 1	LINC00079, MYC, NCRNA00079 onco-lncRNA-100	8q24.21	Type 1 diabetes	Mutation	[78]
				Type 2 diabetes	Mutation	[78, 79]
RNCR3	Retinal non-coding RNA3	LINC00599	8p23.1	Diabetes	Expression	[86]
H19	H19, imprinted maternally expressed transcript	ASM, ASM1, BWS, D11S813E, LINC00008, NCRNA00008,WT2	11p15.5	Type 2 diabetes	Locus	[80]
					Regulation	[81]
PLUT	PDX1 associated lncRNA,upregulator of transcription	PLUTO; HI-LNC71; PDX1-AS1	13q12.2	Type 2 diabetes	Regulation	[87]
MIAT	Myocardial infarction associated transcript	C22orf35, RNCR2, GOMAFU, LINC00066, NCRNA00066, lncRNA-MIAT	22q12.1	Diabetes	Regulation	[88]
				Type 2 diabetes	Expression	[80]
LINC01611	Long intergenic non-protein coding RNA 1611	RP1-90L14.1, TCONS_l2_00025430	6q14.3	Diabetes	Locus	[82]
GAS5	Growth arrest specific 5	NCRNA00030, SNHG2	1q25.1	Type 2 diabetes	Expression	[83]
LINC01370	long intergenic non-protein coding RNA 1370	HILNC25, HI-LNC25	20q12	Type 2 diabetes	Regulation	[84]
LINC00673	Long intergenic non-protein coding RNA 673	HI-LNC75	17q24.3		Locus/ Expression	
LINC01512	Long intergenic non-protein coding RNA 1512	LOC100132354, TCONS_00011120, HI-LNC77	6p21.1		Locus	
LINC01574	Long intergenic non-protein coding RNA 1574	HI-LNC12, TCONS_00009551	5q35.2		Locus	
TUNAR	TCL1 upstream neural differentiation- associated RNA	TUNA, HI-LNC78	14q32.2		Locus	

NOTE: Mutation—Genetic variation in lncRNA genes region on chromosome causes disease and influences susceptibility; Expression—the dysfunction expression of LncRNAs were associated with diabetes; Locus—A genetic susceptibility locus; Regulation—Overexpression or depletion of a LncRNA will regulate other molecules expression to lead to the progression of diabetes. Diabetes—its associated complications, involving in diabetic retinopathy (DR), diabetic nephropathy(DN), diabetic cardiomyopathy (DCM) and diabetic neuropathic pain (DNP).

### Dysregulation and roles of LncRNAs in DM

#### LncRNAs and pancreatic β-cells

Pancreatic islets consist of a class of hormone-releasing cells, and about 70% of pancreatic cells are β-cells. Pancreatic β-cells play a central role in maintaining blood glucose homeostasis through insulin secretion [55, 78]. In turn, insulin controls the carbohydrate and lipid metabolism in the human body. Therefore, reduced secretion of insulin, due to β-cell dysfunction and/or loss, leads to different forms of DM [79]. Recent studies demonstrated that the expression profile of LncRNAs changes spatiotemporally during the maturation process of pancreatic β-cells [73, 76]. Abnormal expression and dysregulation of these LncRNAs, accompanied with the dysfunction of pancreatic β-cells influences cell survival, proliferation, differentiation, or function (especially insulin secretion) and apoptosis. For example, using a whole-genome transcriptome map, Morán et al reported that a series of islet lncRNAs (e.g., HI-LNC25, HI-LNC75, HI-LNC12, and HI-LNC78) were dynamically regulated. They showed that these LncRNAs were not activated in human embryonic pancreatic progenitor cells and were activated in mature pancreatic cells, thus suggesting that these LncRNAs are an integral component of the β cell differentiation and maturation program [73]. Another report demonstrated that a β cell-specific LncRNA, HI-LNC25 downregulates GLIS family zinc finger 3 (GLIS3) mRNA that is an islet transcription factor [80] via a gene regulatory function to influence pancreatic β-cell programing. Moreover, Zou et al. [81] demonstrated that the expression of Sox2 and other stem cell markers (such as Oct4 and Nanog) was downregulated in human amniotic epithelial stem cells (HuAECs) after overexpression of a LincRNA-regulator of reprogramming (LincRNA-ROR) specific siRNA. Meanwhile, miR-145 can play a role in the expression of LncRNA-ROR and Sox2, which is deduced from the results of a luciferase reporter assay. Additionally, LincRNA-ROR effectively maintains Sox2 gene expression through competitive binding with miR-145 to maintain the β islet-like cell differentiation efficiency. During proliferation of pancreatic β-cells, Mutskov et al. [82] showed that the expression of insulin like growth factor 2 antisense RNA (IGF2-AS) was upregulated after β cells were stimulated with high concentration of glucose, thus potentially regulating β cell proliferation. Inflammatory microenvironment is an important factor that results in β cell apoptosis [83]. Motterle et al. [84] showed that the upregulation of mouse islet LncRNA-1 promoted nuclear factor-kappa B (NF-κB) expression that in turn induces the sensitive β cells to undergo apoptosis in pre-diabetic nonobese diabetic (NOD) mice. Taken together, the dysregulation of miRNAs may lead to diabetes mellitus by regulating the biology and functions of pancreatic β-cells.

#### LncRNA and insulin resistance

Insulin resistance is characterised by impaired cellular response to insulin, and the inability of physiological levels of insulin to achieve glucose homeostasis. This pathological state of reduced insulin sensitivity or reactivity in the target tissues or cells is a hallmark of T2DM [18]. The process of insulin resistance is associated with defects in insulin signalling downstream of insulin receptor (INSR) [85], insulin Receptor Substrate 1/2 (IRS-1/2) [86], Phosphoinositide 3-Kinase (PI3K)/AKT Serine/Threonine Kinase (AKT) [87] and Glucose Transporter 4 (GLUT4) [88]. Recently, a growing body of evidence indicates that LncRNAs may act as a link between insulin signalling and insulin resistance. They act as key regulators of gene expression and play significant role in regulating the function of insulin-target tissues, especially liver [89, 90]. Zhu et al. [90] demonstrated that LncRNA maternally expressed gene 3 (MEG3) was upregulated in livers of high-fat diet fed and ob/ob mice. Consequently, they enhanced hepatic insulin resistance via increased expression of forkhead box O1 (FoxO1), a critical regulator of hepatic glucose and lipid metabolism via its ability to regulate the expression of G6pc and Pepck in gluconeogenesis [91]. Moreover, Yan and colleagues [89] reported that the expression of metastasis-associated lung adenocarcinoma transcript 1 (MALAT1) is increased in livers of ob/ob mice, and promoted hepatic insulin resistance by increasing the stability nuclear sterol regulatory element binding transcription factor 1c (SREBP-1c). Therefore, inhibition of the hepatic insulin resistance-associated LncRNAs may be a potential strategy against hyperglycaemia associated with T2DM.

#### LncRNAs and diabetic epigenetic modification

Epigenetics is related to heritable changes in gene expression caused by mechanisms that do not affect the DNA sequence itself. Epigenetic alterations have been repeatedly proposed as a likely molecular mechanism linking birth weight and later determinant of health in childhood or even later in life [92]. Epigenetic modifications can regulate DM-related genes by activating or inhibiting their transcription, thereby affecting glucose homeostasis, β-cell function, insulin secretion and vasculopathy, etc. Moreover, LncRNAs can be localized in the nucleus and participate in the assembly of long noncoding RNA-dependent nuclear bodies by forming chromatin remodelling complexes [93, 94]. An emerging concept suggests that not only LncRNAs regulate gene expression at the epigenetic level, but also their expression could regulated by epigenetic modifications [95]. Epigenetic modifications occur mainly by two most widely studied mechanisms, namely DNA methylation and histone modifications, which are known to be crucial for proper control of gene expression [96, 97]. DNA methylation occurs mainly in sequences enriched in CpG dinucleotides, or the so-called CpG islands that are mainly located in the proximal region of promoters and are underrepresented in the rest of the genome. Methylation of CpG islands is generally associated with gene silencing whereas hypomethylation is linked to hyperactivation of gene expression [79, 96]. A study [98] showed that the abnormal expression of hydatidiform mole associated and imprinted (HYMAI), a special LncRNA that was expressed in paternal alleles [99], is closely associated with transient neonatal diabetes mellitus (TNDM), and such TNDM co-occurs with defective DNA methylation. The DNA methylation at the insulin like growth factor 2 (IGF2)/H19 gene locus, that may be induced by intrauterine hyperglycaemia [100], and is a cornerstone that links birth weight and foetal metabolic programming of late onset obesity [101]. In addition, abnormal DNA methylation of LncRNA MEG3 gene was associated with T1DM [65] and T2DM [102]. However, LncRNAs are also known to regulate DNA methylation. For instance, Dhawan et al. [103] demonstrated that MALAT1 can regulate the level of methyl-CpG binding protein 2 (MeCP2) [104], that plays a role in regulating the methylation of aristaless related homeobox (Arx), to maintain the differentiated status of β cells, and hence, the dynamic equilibrium of glucose. Histone modifications refer to post-transcriptional changes at the amino-terminal of histone subunits. Some histone modifications, such as acetylation, are labile and associated with gene activation, while others, such as methylation, are stable and lead to gene inactivation [79, 96]. For example, Long et al. [105] showed that the LncRNA taurine-upregulated 1 (Tug1) expression is decreased in diabetic podocytes. However, since Tug1 regulates the transcription coactivator PPARγ coactivator 1α (PGC-1α) expression by epigenetically enhancing PGC-1α promoter activity, it influences chronic kidney disease (CKD) development. Meanwhile, Zhuo et al. [106] demonstrated that H19 overexpression induced by administration of lentivirus pcDNA-H19 can inhibit the expression of autophagy-related genes (e.g. LC3-II, ATG7b and BECN1) in diabetic rats. Furthermore, H19 could directly bind to EZH2 in an RNA-binding protein immunoprecipitation (RIP) assay, and the complex of H19/EZH2 could inhibit autophagy by epigenetically silencing the DIRAS family GTPase 3 (DIRAS3) in cardiomyocytes, as seen in a chromatin immunoprecipitation (ChIP) assay. However, the mechanism of and relationship between LncRNAs and diabetic epigenetic modifications is still at an exploratory stage, and the detailed mechanisms still need further research.

### LncRNAs and diabetic complications

#### lncRNA and diabetic nephropathy (DN)

Diabetic nephropathy (DN) is the leading cause of end-stage renal disease (ESRD) in DM, with an incidence of 20%–40%, and is thus a major cause of death and disability in DM [107, 108]. DN is characterized by progressive renal interstitial fibrosis [15] that results in a series of pathological changes, including excessive accumulation of extracellular matrix (ECM), mesangial expansion, thickening of glomerular and tubular basement membranes and increased production of mesangial matrix [109, 110]. Recent studies reported that LncRNAs play a crucial role in the development of DN, and regulate ECM accumulation. Importantly, the gene locus of plasmacytoma variant translocation 1 (PVT1) was reported to be associated with ESRD, which in turn is attributed to T1DM [111] and T2DM [69]. Alvarez et al. [67] reported that PVT1 expression was significantly upregulated in response to glucose treatment in human mesangial cells. The upregulated PVT1 promotes the level of the major ECM proteins, like fibronectin 1 (FN1) and collagen type IV alpha 1 chain (COL4A1), and two key regulators of ECM proteins, namely, transforming growth factor beta 1 (TGFβ1) and plasminogen activator inhibitor-1 (PAI-1). Moreover, they also found that PVT1 acts as a host gene to drive the production of miR-1207-5p which can directly target glucose-6-phosphate dehydrogenase (G6PD), prostate transmembrane protein, androgen induced 1 (PMEPA1), 3-phosphoinositide dependent protein kinase 1 (PDPK1) and SMAD family member 7 (SMAD7), and increase the expression of TGF-β1, PAI-1, and FN1 to regulate the pathogenesis of DN [112]. Furthermore, using miRNA microarray analysis and a luciferase reporter assay, Duan et al. [113] demonstrated that miRNA-377 was remarkably up-regulated in db/db DN mice and LncRNA Tug1 was a direct target of miR-377, respectively. LncRNA Tug1 also acts as an endogenous sponge of miR-377 and downregulates miR-377 expression levels as measured in quantitative real time PCR (qRT-PCR); this relieves the inhibition of its target gene PPARγ, alleviating PAI-1 and TGF-β1 accumulation in mesangial cells (MC). In another report, LincRNA-Gm4419 was shown to participate in NF-κB/NLRP3 inflammasome-mediated inflammation in db/db DN mice [114]. In addition, Zhou et al. [115] demonstrated that LncRNA Myocardial infarction associated transcript (MIAT)- Nrf2 axis may serve as an important signalling pathway for high glucose induced renal tubular epithelial injury, and also in the pathological process of acute kidney injury (AKI).

#### LncRNAs and diabetic retinopathy (DR)

Diabetic retinopathy (DR) is one of the most common complications of DM and a leading cause of catastrophic vision loss in developed nations [116]. DR-induced deterioration of vision is usually accompanied by inflammation, retinal ischemia, neovascularization, vascular hyperpermeability, and vascular cell dysfunction [117, 118]. Therefore, structural or functional abnormality of retinal microvasculature is an important characteristic of DR [119, 120]. Based on the proliferative status of retinal neovasculature, DR can be divided into two categories: non-proliferative diabetic retinopathy (NPDR) and proliferative diabetic retinopathy (PDR) [121, 122]. Through DR has obvious clinical and pathological characteristics, identifying biomarkers to predict DR or to determine therapeutic response is important because of the complex pathogenesis and ambiguous risk factors [123, 124]. Recently, accumulating studies reported that LncRNAs play an important role in the development of DR, especially in retinal microvascular dysfunction. Importantly, MALAT1 was the earliest reported aberrantly expressed LncRNA in DR [125], and first identified to be crucial for the angiogenic response of endothelial cells as well as for vascularization *in vivo* [120, 126]. Soon afterwards, a series of LncRNAs have been reported to be associated with DR [127], such as retinal non-coding RNA3 (RNCR3) [128, 129], MIAT [77, 130], MEG3 [131], etc. Interestingly, the regulatory mechanism involving LncRNA-miRNA-mRNA manner. For example, Shan et al. [128, 129] revealed that LncRNA RNCR3 is significantly up-regulated in diabetic mice or in RF/6A cells, which were exposed to high glucose. This was measured by qRT-PCR. RNCR3 can act as ceRNA with miR-185-5p to regulate its target gene kruppel-like factor 2 (KLF2) as observed by using a luciferase reporter assay and the gene knockdown technique to silence the expression of RNCR3. The RNCR3/miR-185-5p/KLF2 complex was also involved in atherosclerosis and DM-induced retinal microvascular abnormalities. Moreover, LncRNA MIAT has been related closely with DM-induced retinal microvascular dysfunction *in vivo* and *in vitro*, via its interaction with miR-150-5p [77] and miR-29b [130]. Furthermore, LncRNAs are able to take part in regulating retinal ganglion cell (RGC) injury, which is another important pathological feature of DR [132]. Li et al. [133] demonstrated that LncRNA SOX2 overlapping transcript (LncRNA-Sox2OT) knockdown can protect RGCs against high glucose-induced injury and play neuroprotective role in DM-related retinal neurodegeneration *in vivo*. Meanwhile, Liu et al. [75] revealed that RNCR3 knockdown alleviates DM-induced retinal neurodegeneration by reducing the expression of glial reactivity-related genes including glial fibrillary acidic protein (GFAP) and vimentin. These studies suggest LncRNAs have a strong potential to act as therapeutic targets for treating DR.

#### LncRNAs and diabetic cardiomyopathy (DCM)

Diabetic cardiomyopathy (DCM) is one of the prominent cardiovascular complications of DM, and carries a substantial risk for the subsequent development of heart failure and increased mortality [134]. DCM is defined as myocardial dysfunction occurring in patients with diabetes in the absence of coronary artery disease, hypertension, or valvular heart disease [135, 136]. The pathogenesis of DCM may involve inflammation, oxidative stress, mitochondrial dysfunction, impaired calcium handling, renin-angiotensin system activation, cardiomyocyte apoptosis [137]. Recently, increasing evidence demonstrated that LncRNAs play a crucial role in regulating cardiac muscle [138], such as growth arrest-specific 5 (GAS5) [139], cardiac mesoderm enhancer-associated noncoding RNA (CARMEN) [140], urothelial carcinoma-associated 1 (UCA1) [141],etc. However, currently only two lncRNAs have been associated with DCM, namely, MALAT1 [142, 143], and H19 [106, 144]. MALAT1 expression is upregulated in patients with myocardial infarction (MI) [58] and in cardiac tissue of diabetic rats [142, 143]. Two studies by Zhang and colleagues [142, 143] revealed that MALAT1 knockdown can reduce diabetes-induced myocardial inflammation as well as cardiomyocyte apoptosis, and consequently improve left ventricular function in diabetic rats. Compared with MALAT1, the regulatory mechanism of H19 in DCM was shown to be more complex. The H19 LncRNA encodes a 2.6 kb capped, spliced and polyadenylated noncoding RNA that is predominantly cytoplasmic, with a minor fraction being localised in the nucleus [145]. However, H19, that is downregulated by acute hyperinsulinemia [70], can exert its biological function in the nucleus by binding and recruiting the histone methyltransferase EZH2 at the promoter of DIRAS3. EZH2 recruitment in turn leads to the epigenetic silencing of DIRAS3, and therefore inhibiting DIRAS3-induced autophagy, and protecting cardiomyocytes exposed to high glucose [106]. In an alternative mechanism, cytoplasmic H19 can act as miRNA sponge to sequester miR-106a as well as the miR-let7 family members [146, 147]. Moreover, H19 can serve as a precursor of miR-675 that will in turn, post-transcriptionally regulate a number of target genes involved in cell proliferation and differentiation [52, 148]. Zhang et al. [144] demonstrated that miR-675 expression was decreased after transfection with H19 siRNA in cardiomyocytes. As shown by a luciferase reporter assay, miR-675 can directly target the voltage-dependent anion channel 1 (VDAC1), thereby forming the H19/miR-675/VDAC1 axis to inhibit apoptosis induced by high glucose. This finding may provide a novel therapeutic strategy for the treatment of DCM.

#### LncRNAs and diabetic neuropathic pain (DNP)

Diabetic neuropathic pain (DNP) is one of the most common chronic complications of T2DM, fifty percent of diabetic patients suffer from DNP [149]. Typical symptoms of DNP include pathological nerve pain, including spontaneous pain, hyperalgesia (increased pain perception to noxious stimuli), and allodynia (pain to normally innocuous stimuli) [149, 150]. Patients with DNP are predominantly characterized by sensory symptoms like “glove-and-stocking” distribution and have burning/ lancing (stabbing) sensations, tingling (“pins and needles” or paraesthesia), or shooting (electric shock) [149, 150]. DNP has become a substantial problem in the field of intractable pain therapy [151], which is frequently accompanied by diminished quality of life in diabetic patients [152]. Recently, a few studies show that LncRNAs play a crucial role in progress of DNP. LncRNA-NONRATT021972, a blood glucose regulator [153], is a typical representative associated with DNP. On one hand, NONRATT021972 was showed to be related with diabetic autonomic neuropathy (DAN) [154] by Xu and colleagues [155]. Their study reported that NONRATT021972 induces the expression of tumour necrosis factor-α (TNF-α) and serine phosphorylation of insulin receptor substrate 1 (IRS1) in superior cervical ganglia (SCG) to mediate heart rate variability (HRV) in diabetic rats. Moreover, NONRATT021972 is associated with the abnormalities of function in the dorsal root ganglia (DRG) [149] that is a clinical characteristic of DNP. Liu et al. [156] reported that NONRATT021972 siRNA treatment can decrease the expression levels of purinergic receptor P2X 7 (P2X_7_) and inflammatory factors TNF-α to inhibit the excitability of DRG neurons, reducing mechanical and thermal hyperalgesia in T2DM rats. Moreover, Peng et al. [157] demonstrated that NONRATT021972 is a potential induction factor to promote the development of DNP via the P2X_3_ receptor expression in rat DRG. In another study, LncRNA uc.48+ was also reported to mediate the P2X_3_ receptor expression, thereby promote the excitatory transmission in DRG [158]. Overall, these studies provide novel insights for the treatment of DNP.

#### LncRNAs and diabetic other complications associated with DM

LncRNAs also display a role other complications associated with DM. For instance, in the aspects of inflammatory complications of DM, Reddy et al. [159] reported that lncRNA-E330013P06 was upregulated in macrophages from db/db mice and T2DM patients, and induced the expression of several genes to heighten the inflammatory response. In addition, Puthanveetil et al. [160] demonstrated that glucose stimulates human umbilical vein endothelial cells (HUVECs) and can increase the expression of MALAT1 and serum amyloid A 3 (SAA3) as measured in a qRT-PCR. The expression of MALAT1, SAA3 and inflammatory mediators IL-6 and TNF-α were down-regulated after transfection of the MALAT1-specific siRNA, which may eventually promote diabetes-induced micro and macrovascular complications. Further, antisense noncoding RNA for the INK4 locus (ANRIL) was shown to upregulate vascular endothelial growth factor (VEGF) expression and promote angiogenesis by activating NF-κB inflammation signalling pathway in DM combined cerebral infarction (CI) rats [161]. In the aspects of diabetes-associated cognitive impairment, Li et al. [162] demonstrated that PVT1-mediated autophagy may protect hippocampal neurons from impairment of synaptic plasticity and apoptosis, and then ameliorate cognitive impairment in streptozotocin (STZ)-induced diabetic mice with the cognitive impairment induced by 3-methyladenine (3-MA). In the aspects of diabetes-associated abnormal immunity, LncRNAs show a potential regulatory function in T1DM. T1DM is an organ-specific autoimmune disease characterized by chronic and progressive apoptotic destruction of pancreatic β cells. Research in recent years indicates that LncRNAs modulate the apoptosis of sensitive β cell and they may emerge as novel biomarkers in diagnosis and as targets for development of new therapies for T1DM [84, 163].

#### Potential clinical applications of LncRNAs in DM

Through the tremendous progress in the therapy of DM, the morbidity and mortality caused by DM is still high in worldwide. Inspirationally, numerous studies showed that the therapeutic efficacy for DM patients could be improved via the early detection of DM, followed by timely intervention with particular attention to blood pressure control (thus limiting proteinuria), glycaemic control and cardiovascular risk control, etc. [23, 24]. Therefore, there is a great urgency to prevent and treat diabetes by searching for novel molecular markers and drug targets. With the application of next-generation sequencing in RNA-seq technology [164], changes in the expression of LncRNAs have been found to be prevalent in the progress of DM and its associated complications. Because of their unique structure, LncRNAs are more stable in a disease, tissue, cell, or development phase specific manner [45]. Further, LncRNAs are easy to extract and detect with higher specificity compared with proteins. Detecting LncRNAs by RT-PCR and in-situ hybridization is also more specific and sensitive than detecting proteins by an antigen-antibody reaction [165]. There is also a greater possibility to screen for sensitive and specific molecular biomarkers from thousands of LncRNAs with regard to the relatively small number of miRNAs.

Recent substantial evidence indicates that the expression of many LncRNAs varies in normal/diabetic animal models [125, 154, 166] and healthy/diabetic patients’ clinical files [69, 72]. Therefore, LncRNAs have served as novel potential biomarkers for diagnosis and prognosis of DM. For example, GAS5 has been proposed as a prognostic biomarker [72], receiver operating characteristic (ROC) curve analysis revealed that GAS5 expression was a suitable candidate to distinguish between diabetic and non-diabetic samples (sensitivity 85.1%, specificity 67.3%). The area under curve (AUC) of ROC were 0.81 [OR (Odds Ratio) = 11.79, 95% confidence interval (CI): 3.97, 37.26), *p* < 0.001], respectively (Table 2). In conclusion, the use of LncRNAs outweighs the use of current DM biomarkers in many fields, and they could be promising molecular biomarkers and new targets for future drugs. LncRNAs, novel molecules that recently come to our attention, are no longer considered anomalies or noise. On the contrary, they have become a popular research subject, after siRNAs and miRNAs, owing to their important functions [150]. We believed that further investigation into the characteristic and function of LncRNAs will be of great value in preventing and treating diabetes.

**Table 2:** Application index of LncRNA in human DM

LncRNAs	Diabetes Type	Sample numbers (non-diabetic/diabetic)	AUC	Sensitivity	Specificity	OR (95%CI*)	Ref.
GAS5	Type 2 diabetes	49/47	0.81	85.1%	67.3%	11.79 (3.97, 37.26)	[83]

## CONCLUSIONS AND PROSPECTIVE

**Figure 2:**
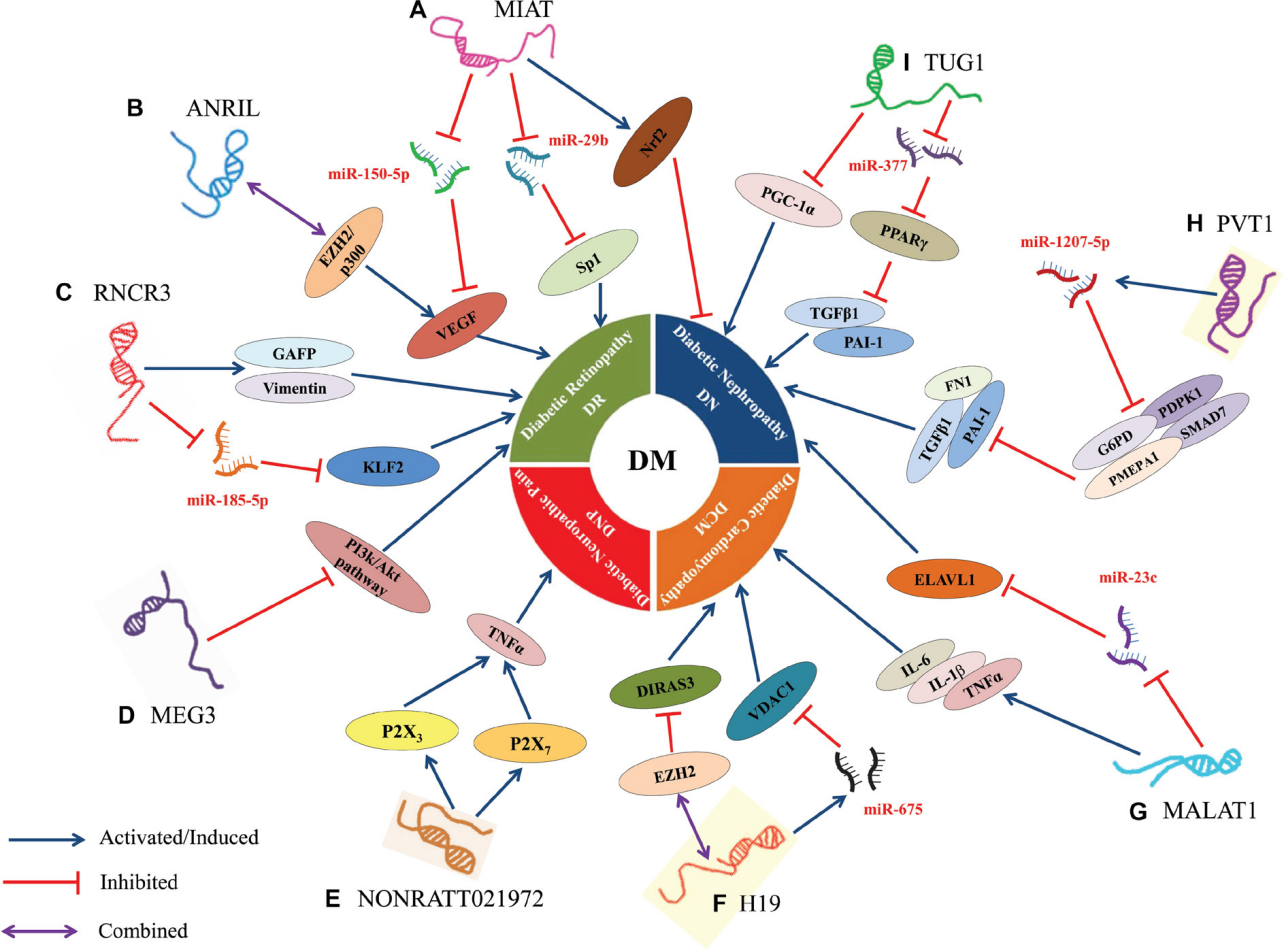
Dysregulation and functional roles of LncRNAs in DM (**A**) MIAT regulated microvascular dysfunction via a ceRNA regulatory network MIAT/miR-150-5p/VEGF in diabetic retinas and endothelial cells; MIAT promoted cell apoptosis by absorbing miR-29b and subsequently relieving its repressive effect on the targeting genes Sp1 in high glucose stimulated rat retinal Müller cells (rMC-1); MIAT suppressed high glucose-induced tubular damage by promoting the expression of Nrf2 in human renal tubular epithelial cell line (HK-2). (**B**) ANRIL regulated VEGF expression prompted the development of DN by directly binding the p300 and EZH2 complex. (**C**) RNCR3 promoted DM-induced retinal neurodegeneration because of retinal cell apoptosis and Müller glial cell proliferation via the increased expression of glial reactivity-related genes including GFAP and vimentin; RNCR3 regulated endothelial cell function through RNCR3/miR-185-5p/KLF2 regulatory network to promote DM-induced retinal microvascular abnormalities. (**D**) MEG3 inhibited retinal endothelial cell proliferation, migration, and tube formation by suppressing the activation of PI3k/Akt signalling in the retinas of STZ-induced diabetic mice. (**E**) NONRATT021972 could interact with P2X_3_ or P2X_7_ to induce the release of inflammatory factors (TNF-α), thereby resulting in the excitability of DRG neurons to increase the risk of DNP. (**F**) H19 inhibited autophagy in cardiomyocytes exposed to high glucose by recruiting EZH2 and subsequently suppressing DIRAS3 expression; H19, a host gene of miR-675, also suppressed high glucose-induced cardiomyocyte apoptosis by reducing the expression of miR-675 target gene VDAC1. (**G**) MALAT1 attenuated the diabetes-induced myocardial inflammation in myocardial tissue of diabetic rats by reducing the levels of inflammatory markers such as TNF-α, IL-1β and IL-6; MALAT1 facilitated renal tubular epithelial pyroptosis by modulated miR-23c targeting of ELAVL1 in STZ-induced diabetic rats. (**H**) PVT1, a host gene of miR-1207-5p, suppressed the expression of miR-1207-5p targeting genes (G6PD, PMEPA1, PDPK1, and SMAD7), thereby increasing the expression of ECM-related genes FN1, PAI-1 and TGF-β1 in mesangial cells. (**I**) TUG1 regulated the metabolism of DN by epigenetic targeting of expression of PGC-1α in podocytes; TUG1 suppressed ECM accumulation via TUG1/miR-377/PPARγ signalling, and was also involved in reciprocally suppressing miR-377 and increasing the expression of targeted gene PPARγ to decrease the ECM accumulation in mesangial cell. In this diagram, the symbol “
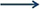
”represents activated or induced; the symbol “
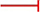
”represents inhibited; and the symbol “
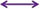
” represents combined or sponged.

To summarize, increasing studies showed that LncRNAs have close connection with the progression of DM and its associated complications, such as pancreatic β-cells disorder, insulin resistance and epigenetic regulation, *etc.*, by forming a complex regulatory network (Figure 2). LncRNAs, as novel regulatory biomarkers, participate in multiple steps of DM by modulating the expression of related-genes at the epigenetic, transcriptional and posttranscriptional levels. According to the role and features of DM-related LncRNAs, we may silence or active the LncRNAs in DM patients via an exogenous means (e.g., gene knock in, RNA interference and gene supplement) to achieve the prevention and treatment of DM. Although many important biological functions of lncRNAs have been discovered during the past three years, an overwhelming majority of LncRNAs have not been well-characterized, and there is still a long to go before we can identify, characterize, and elucidate the actual functions of LncRNAs in the pathogenesis of DM at the molecular level and use them in clinical practice and for therapeutic intervention. Currently, the research of LncRNAs faces two challenges. On one hand, there is need to sequentially validate the LncRNAs derived from the RNA sequencing in human tissue or cells to demonstrate whether they have function. In addition, there is a need to explore the functional LncRNAs to establish whether they are associated with specifically one or more diseases and by what molecular mechanisms. Although our current knowledge on LncRNAs is only the tip of an iceberg, novel methods and technology will eventually lift the mysterious veil covering LncRNAs, thereby providing a novel strategy for the prevention, early diagnosis and treatment of DM.

### Highlights

LncRNAs have emerged as an essential regulator in almost every aspect of biology, Misexpression of LncRNAs alters the progression of diabetes mellitus(DM) and its associated complications, involving pancreatic β-cell disorder, insulin resistance, epigenetic regulation, *etc*. LncRNAs serve as a promising target for DM prevention, early diagnosis, and therapy.

## Abbreviations

LncRNAs: long non-coding RNAs
ORF: open reading frame
DM: diabetes mellitus
HGP: human Genome Project
siRNA: small interfering RNA
NCD: non-infective diseases
RMRP: RNA component of mitochondrial RNA processing endoribonuclease
DDX5: DEAD-box helicase 5
RORγ: Retinoid-related orphan receptor gamma
EZH2: enhancer of zeste homolog-2
HDAC1: histone deacetylase 1
Dnmt3a: DNA-methyltransferase 3 alpha
PACER: p50-associated COX-2 extragenic RNA
COX2: cytochrome c oxidase subunit II
Linc-MD1: Long intergenic non-protein coding RNA, muscle differentiation 1
ceRNA: competing endogenous lncRNA
T1DM: type 1 diabetes mellitus
T2DM: type 2 diabetes mellitus
DN: diabetic nephropathy
DR: diabetic retinopathy
DCM: diabetic cardiomyopathy
DNP: diabetic neuropathic pain
GLIS3: GLIS family zinc finger 3
LincRNA-ROR: LincRNA-regulator of reprogramming
IGF2-AS: insulin like growth factor 2 antisense RNA
NOD: nonobese diabetic
NF-κB: nuclear factor-kappa B
INSR: insulin receptor
IRS-1/2: insulin receptor substrate 1/2
PI3K: phosphoinositide 3-Kinase
AKT: AKT Serine/Threonine kinase
GLUT4: glucose transporter 4
MEG3: maternally expressed gene 3
FoxO1: forkhead box O1
MALAT1: metastasis-associated lung adenocarcinoma transcript 1
SREBP-1c: sterol regulatory element binding transcription factor 1c
HYMAI: hydatidiform mole associated and imprinted
TNDM: transient neonatal diabetes mellitus
IGF2: insulin like growth factor 2
MeCP2: methyl-CpG binding protein 2
Arx: aristaless related homeobox
Tug1: taurine-upregulated 1
PGC-1α: PPARγ coactivator 1α
CKD: chronic kidney disease
DIRAS3: DIRAS family GTPase 3
ESRD: end-stage renal disease
ECM: extracellular matrix
PVT1: plasmacytoma variant translocation 1
FN1: fibronectin 1
COL4A1: collagen type IV alpha 1 chain
TGFB1: transforming growth factor beta 1
PAI-1: plasminogen activator inhibitor-1
G6PD: glucose-6-phosphate dehydrogenase
PMEPA1: prostate transmembrane protein, androgen induced 1
PDPK1: 3-phosphoinositide dependent protein kinase 1
SMAD7: SMAD family member 7
MIAT: myocardial infarction–associated transcript
AKI: acute kidney injury
NPDR: non-proliferative diabetic retinopathy
PDR: proliferative diabetic retinopathy
RNCR3: retinal non-coding RNA3
KLF2: kruppel like factor 2
RGC: retinal ganglion cell
LncRNA-Sox2OT: LncRNA SOX2 overlapping transcript
GFAP: glial fibrillary acidic protein
GAS5: growth arrest-specific 5
CARMEN: cardiac mesoderm enhancer-associated noncoding RNA
UCA1: urothelial carcinoma-associated 1
MI: myocardial infarction
VDAC1: voltage dependent anion channel 1
DAN: diabetic autonomic neuropathy
TNF-α: tumor necrosis factor α
IRS: insulin receptor substrate
SCG: superior cervical ganglia
HRV: heart rate variability
DRG: dorsal root ganglia
P2X_7_: purinergic receptor P2X 7
SAA3: serum amyloid A 3
HUVECs: human umbilical vein endothelial cells
ANRIL: antisense noncoding RNA in the INK4 locus
VEGF: vascular endothelial growth factor
3-MA: 3-methyladenine
STZ: Streptozotocin
ROC: receiver operating characteristic
AUC: area under curve
OR: Odds Ratio
CI: confidence interval
